# Supplementary material for: Structural convergence endows nuclear transport receptor Kap114p with a transcriptional repressor function toward TATA-binding protein
Source: Nat Commun. 2023 Sep 8;14:5518. doi: 10.1038/s41467-023-41206-9 (PMC10491584; doi:10.1038/s41467-023-41206-9)
Supplement: Supplementary file 1 — Supplementary Information [file 41467_2023_41206_MOESM1_ESM.pdf]

Supplementary Information

Structural convergence endows nuclear transport receptor Kap114p with a transcriptional repressor function toward TATA-binding protein

Chung-Chi Liao[1,2], Yi-Sen Wang[2], Wen-Chieh Pi[3], Chun-Hsiung Wang[4], Yi-Min Wu[4], Wei-Yi Chen[3,5,6] and Kuo-Chiang Hsia[1,2,3,6]

[1]Molecular and Cell Biology, Taiwan International Graduate Program, Academia Sinica and National Defense Medical Center, Taipei 11490, Taiwan
[2]Institute of Molecular Biology, Academia Sinica, Taipei 11529, Taiwan
[3]Institute of Biochemistry and Molecular Biology, College of Life Sciences, National Yang Ming Chiao Tung University, Taipei 11221, Taiwan
[4]Institute of Biological Chemistry, Academia Sinica, Taipei 11529, Taiwan
[5]Cancer and Immunology Research Center, National Yang Ming Chiao Tung University, Taipei, Taiwan

[6]Correspondence should be addressed to K.-C. H. (e-mail: khsia@gate.sinica.edu.tw) and W.-Y. C. (e-mail: chenwy@nycu.edu.tw)

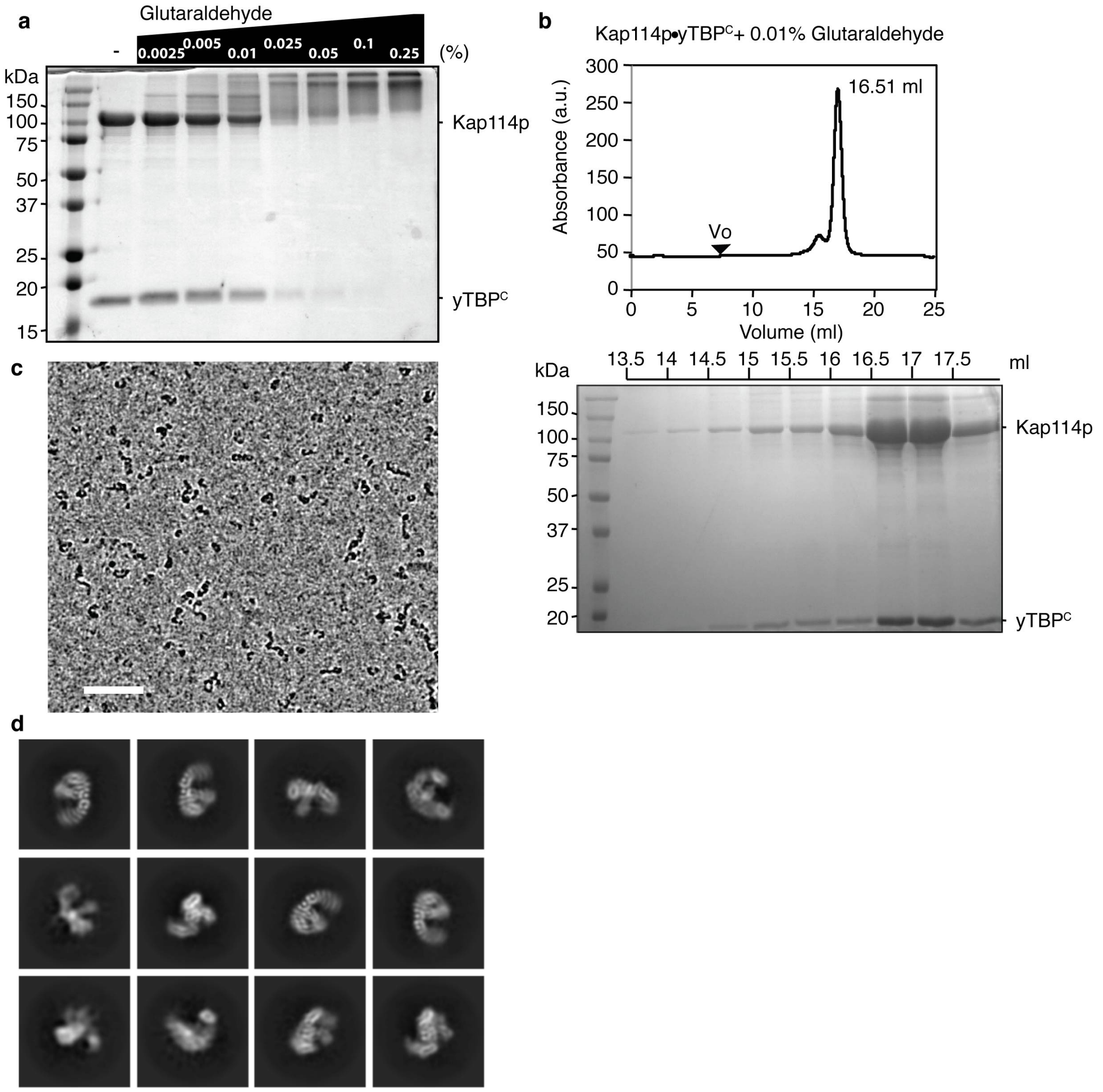

Supplementary Figure 1|Biochemical characterization of purified Kap114p•yTBP^C complex. a, Purified Kap114p•yTBP^C complex was cross-linked with indicated concentrations of glutaraldehyde, analyzed by SDS-PAGE and stained with Coomassie blue. **b**, Kap114p•yTBP^C complex cross-linked by 0.01% glutaraldehyde was anaylzed by size exclusion chromatography (SEC). The SEC (Superdex 200) elution profile of the Kap114p•yTBP^C complex is shown in the upper panel. Peak fractions were analyzed by SDS-PAGE and stained with Coomassie blue. The void volume (*Vo*) of the peak fraction and absorbance (a.u.) at 280 nm for each complex are indicated. **c**, A representatve cryo-electron micrograph of the Kap114p•yTBP^C particles. A field of Kap114p•yTBP^C particles adsorbed onto a

glow-discharged carbon grid and processed for imaging. Scale bar, 35 nm. **d**, Representative reference-free 2D class-average images of the Kap114p•yTBP^{C} particles.

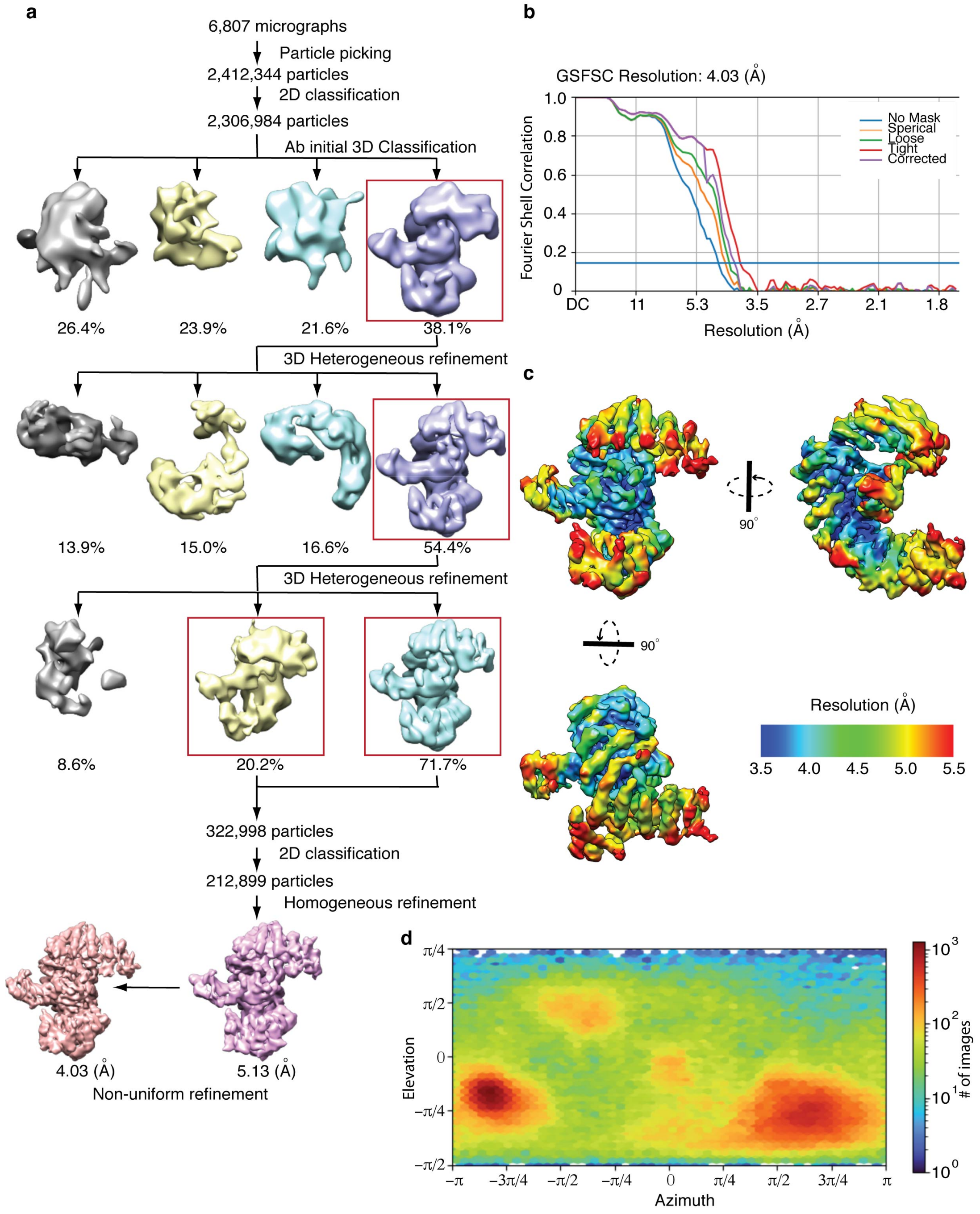

Supplementary Figure 2|Cryo-EM results of the Kap114p•yTBP^{C} complex. a, Overview of cryo-EM data processing pipeline for the Kap114p•yTBP^{C} complex. The micrographs were first processed using Relion and MotionCor2[1,2]. Motion-corrected micrographs were then imported into cryoSPARC[3] for further single-particle reconstruction. 2D classification, *ab initio* model generation, and 3D refinement were conducted using cryoSPARC. **b**, The final resolution obtained from processing 212,899 particles, reported according to gold-standard Fourier Shell

Correlation (FSC) with a cut-off of 0.143. **c**, The cryo-EM map of the Kap114p•yTBP^{C} complex, colored based on local resolution. Three rotated views of the map are shown. **d**, The angular plot of the orientations calculated in cryoSPARC and assigned to the 212,899 particles. The Heat map shows number of particles for each viewing angle.

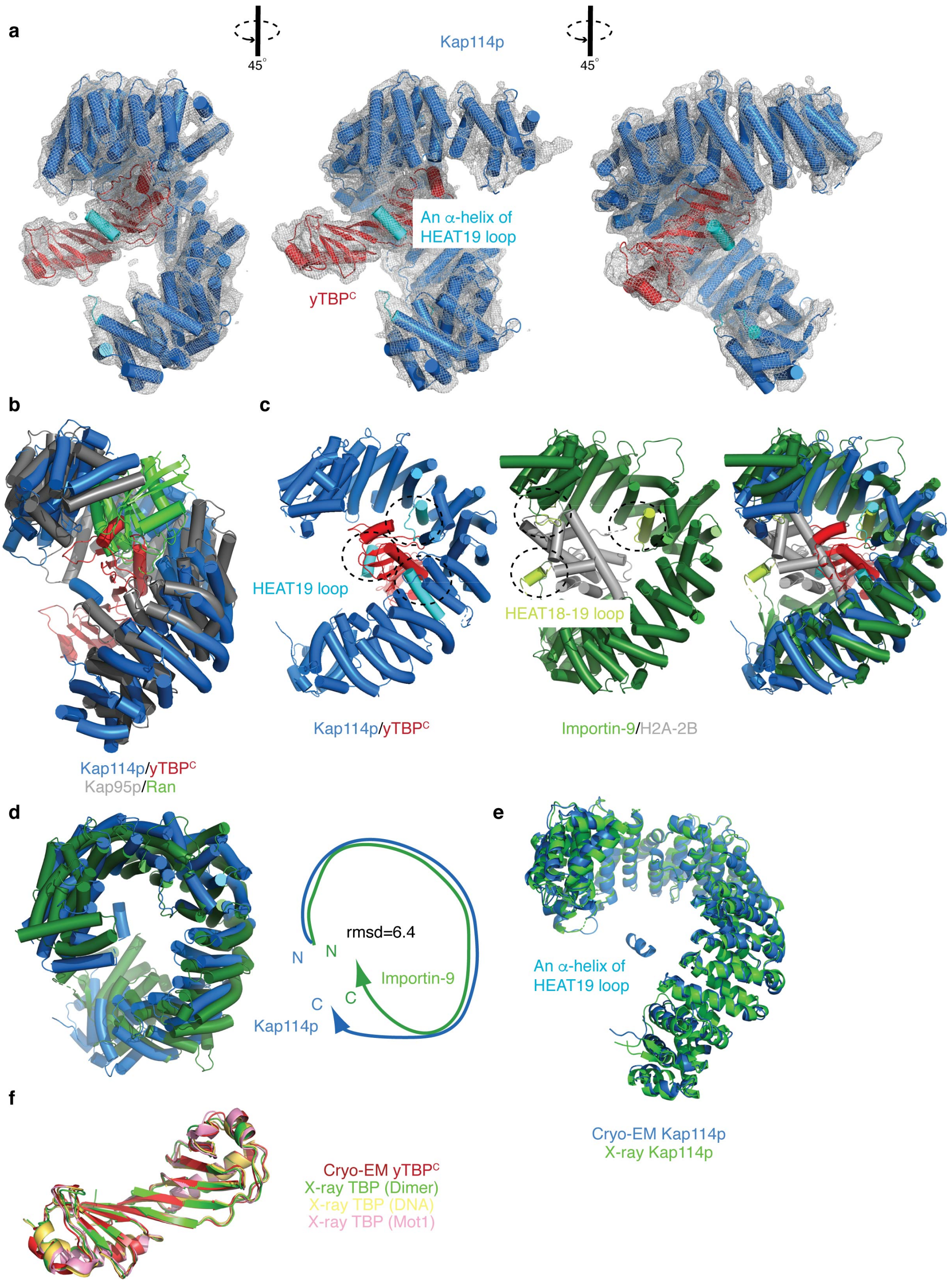

Supplementary Figure 3|Structural comparison of the Kap114p•yTBP^{C} complex with other TBP-containing complexes. a, Crystal structures of Kap114p (blue; PDB code: 6AHO) and yTBP^{C} (red; PDB code: 1YTB) were docked into the corresponding electron microscopy density map colored in transparent gray. Three rotated views are shown. An α-helix in the HEAT19 loop of Kap114p is shown in light blue. **b**,

Superimposition of the cryo-EM structure of Kap114p•yTBP^{C} with the crystal structure of Kap95p•Ran (PDB code: 2BKU) in cartoon representation. Helices are drawn as cylinders and color-coded as indicated. **c**, Superimposition of the Kap114p•yTBP^{C} complex with the crystal structure of Importin-9•H2A-H2B (PDB code: 6N1Z) in cartoon representation. Interacting surfaces of the Kap114p•yTBP^{C} and Importin-9•H2A-H2B complexes are colored light blue or light green, respectively, and highlighted by dashed circles. The HEAT19 loop and HEAT18-19 loop mediating cargo binding by the Kap114p•yTBP^{C} and Importin-9•H2A-H2B complexes, respectively, are color-coded as indicated. **d**, Superimposition of yTBP^{C}-bound Kap114p (blue) with H2A-H2B-bound Importin-9 (green) in cartoon representations (left panel). A schematic representation of the two structures with respective rmsd values is also shown (right panel). **e**, Superimposition of the Kap114p structures determined by X-ray crystallography (green) and cryo-EM (blue) in cartoon representations. **f**, Superimposition of the TBP structures in complex with Kap114p (red), TBP (green; PDB code: 1TBP), DNA (yellow; PDB code: 1YTB) and Mot1 (pink; PDB code: 3OC3) in cartoon representations.

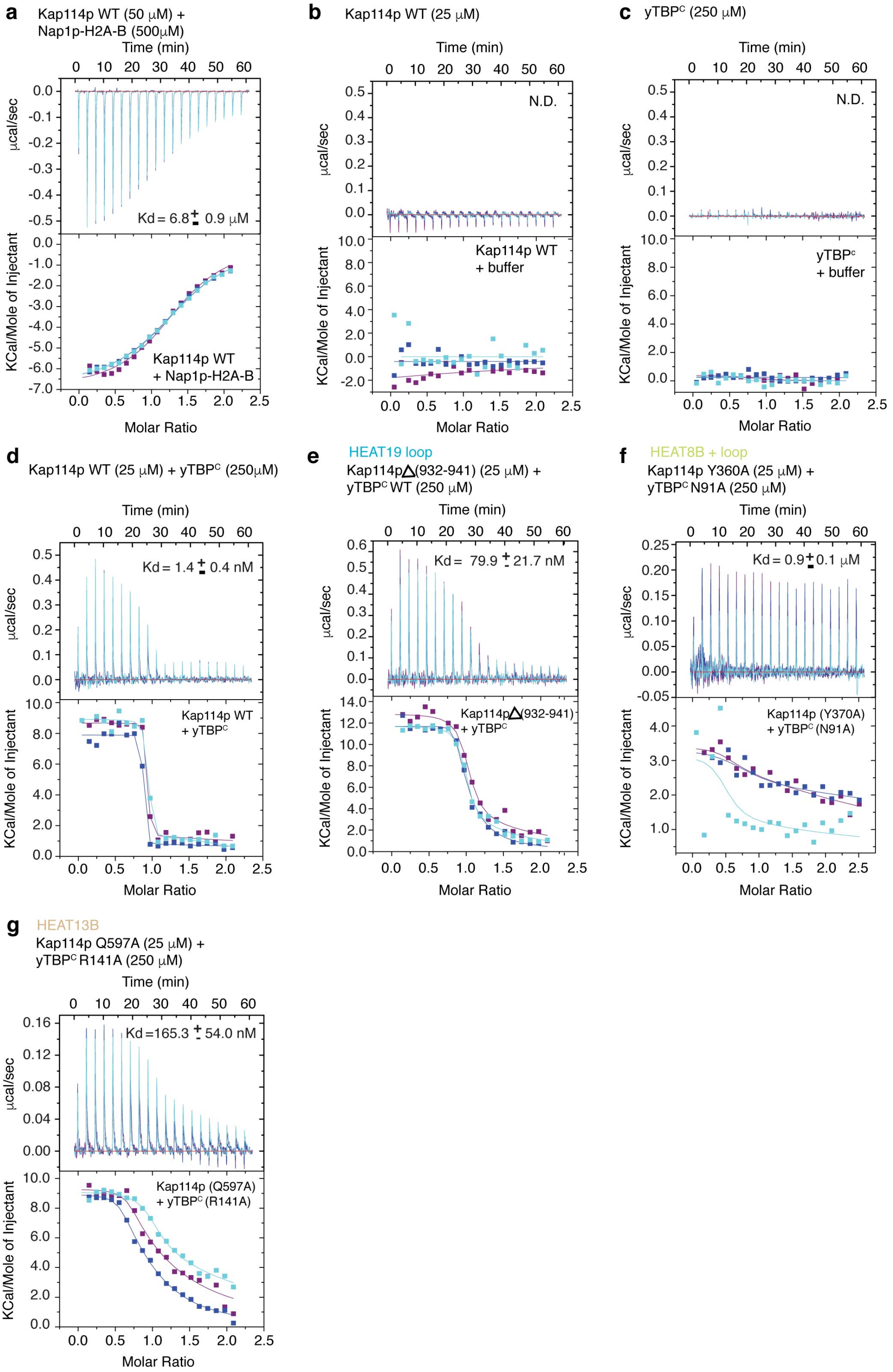
a
Kap114p WT (50 μM) +
Nap1p-H2A-B (500μM)
Kd = 6.8 ± 0.9 μM
Kap114p WT
+ Nap1p-H2A-B
b
Kap114p WT (25 μM)
N.D.
Kap114p WT
+ buffer
c
yTBPᶜ (250 μM)
N.D.
yTBPᶜ
+ buffer
d
Kap114p WT (25 μM) + yTBPᶜ (250μM)
Kd = 1.4 ± 0.4 nM
Kap114p WT
+ yTBPᶜ
e
HEAT19 loop
Kap114pΔ(932-941) (25 μM) +
yTBPᶜ WT (250 μM)
Kd = 79.9 ± 21.7 nM
Kap114pΔ(932-941)
+ yTBPᶜ
f
HEAT8B + loop
Kap114p Y360A (25 μM) +
yTBPᶜ N91A (250 μM)
Kd = 0.9 ± 0.1 μM
Kap114p (Y370A)
+ yTBPᶜ (N91A)
g
HEAT13B
Kap114p Q597A (25 μM) +
yTBPᶜ R141A (250 μM)
Kd = 165.3 ± 54.0 nM
Kap114p (Q597A)
+ yTBPᶜ (R141A)
Time (min)
μcal/sec
KCal/Mole of Injectant
Molar Ratio

Supplementary Figure 4|Binding affinity between Kap114p and its cargoes. a, ITC titration curves (upper) and binding isotherms (lower) of Kap114p with Nap1p-H2A-H2B. **b,c,** ITC titration curves (upper) and binding isotherms (lower) of control buffer with Kap114p (**b**) or yTBP^{C} (**c**). The Kd value is indicated. Data are represented as mean ± SD (n=3 independent experiments). **d-g**, ITC titration curves (upper) and binding isotherms (lower) of WT Kap114p with WT yTBP^{C} (**d**), Kap114pΔ(932-941) with WT yTBP^{C} (**e**) , Kap114p (Y370A) with yTBP^{C} (N91A) (**f**), and Kap114p (Q597A) with yTBP^{C} (R141A) (**g**). Protein concentrations and Kd values are indicated. Data are represented as mean ± SD (n=3 independent experiments).

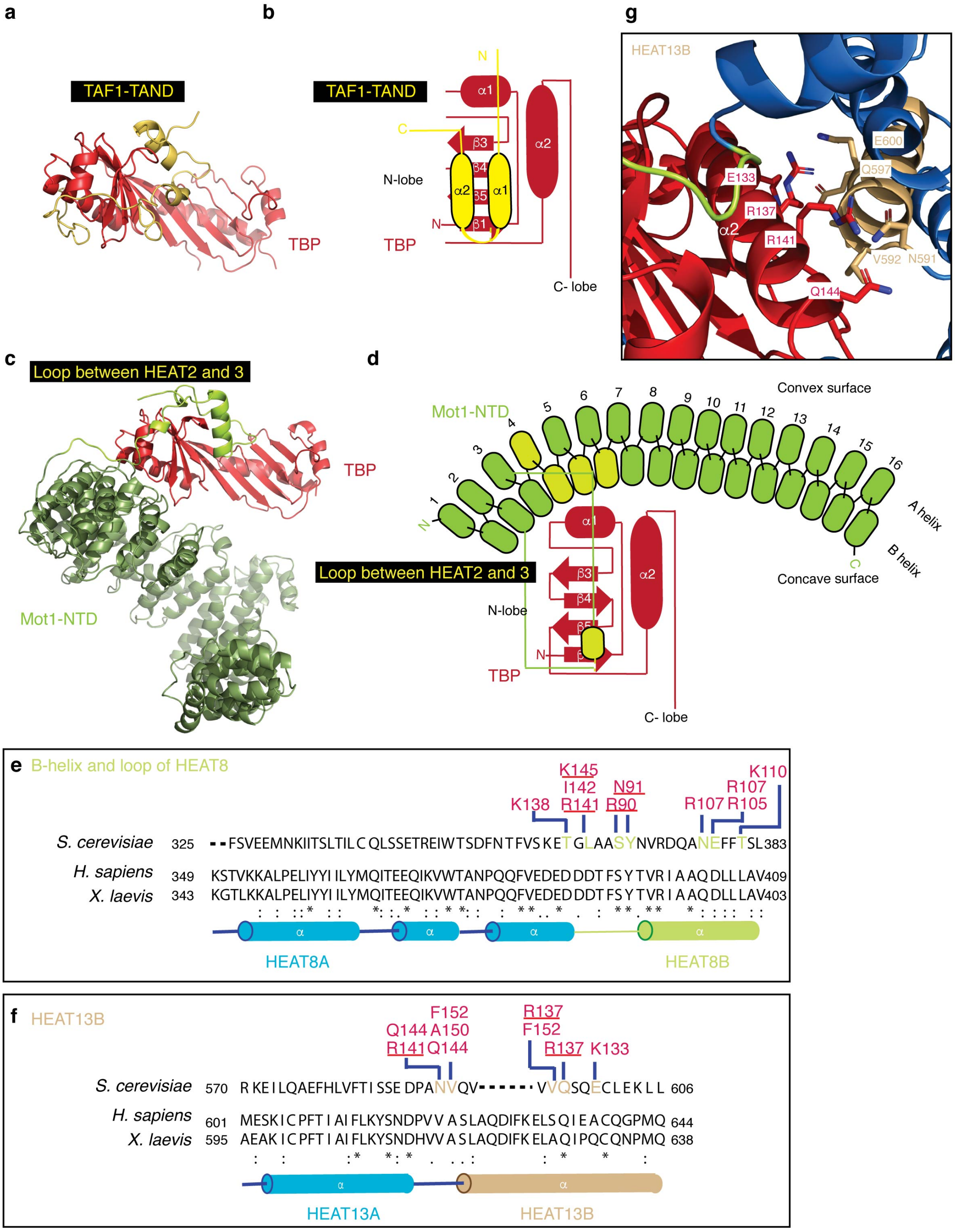

Supplementary Figure 5|Crystal structures of the Mot1-NTD•*Ec*TBP and TAF1-TAND•yTBP complexes. a, Cartoon representation of the Mot1-NTD•*Ec*TBP structure. Two rotated reviews are shown. Mot1-NTD and *Ec*TBP are shown in green and red, respectively. Binding surfaces on Mot1-NTD that contribute to the *Ec*TBP interaction are highlighted in light green. A loop between HEAT2 and 3 that binds to

the concave surface of *Ec*TBP is indicated. **b**, Schematic representation of the domain configuration of Mot1-NTD using the same color code as shown in B. Only the N-lobe of *Ec*TBP is shown in red. **c**, Cartoon representation of the TAF1-TAND•yTBP structure. Two rotated reviews are shown. TAF1-TAND and yTBP are shown in yellow and red, respectively. **d**, Schematic representation of the domain configuration of TAF1-TAND using the same color code as shown in D. Only the N-lobe of yTBP is shown in red. **e**,**f**, Protein sequence alignments (by Clustal Omega[4]) of the B-helix and loop of HEAT8 (**e**) and HEAT13B (**f**) from indicated species (numbers represent amino acid positions). * indicates fully conserved residues; : indicates residues showing strongly similar properties; . represents residues displaying weakly similar properties. An α-helix was assigned to a segment of the amino acid sequence based on protein secondary structure prediction (red, as assessed in PSIPRED[5]) and is depicted as a light blue cylinder. Residues involved in the inter-molecular interaction network of Kap114p (light blue) in complex with $yTBP^{C}$ (red) are indicated. Blue lines indicate interaction residues. **g,** A different angle of Fig. 4b highlights the interactions of HEAT13B of Kap114p with $yTBP^{C}$. Residues mediating Kap114p and $yTBP^{C}$ are indicated. $yTBP^{C}$ (red) and Kap114p (blue) are shown as a stick with line side-chain and cartoon representations, respectively. HEAT13B is colored light orange.

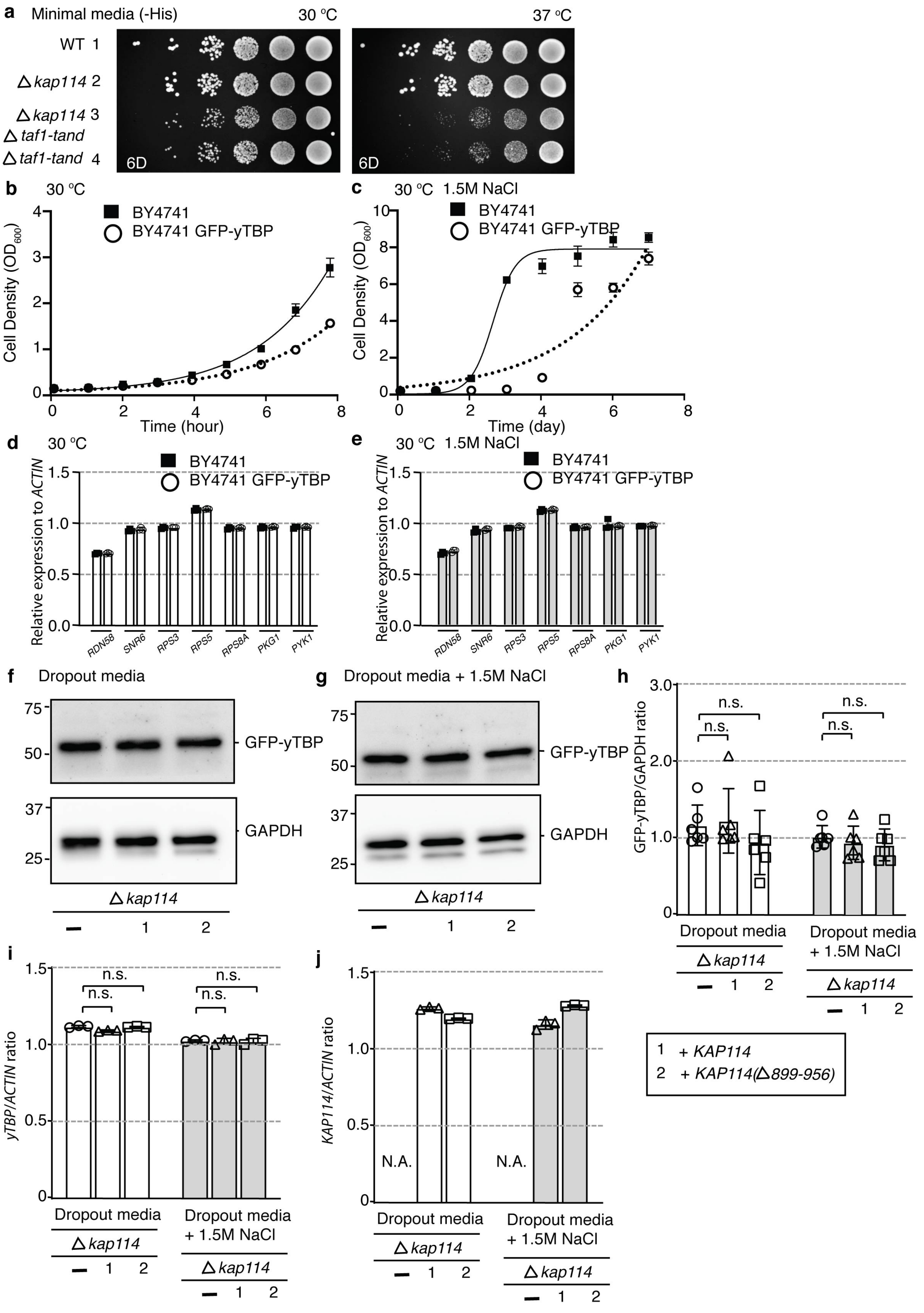

Supplementary Figure 6|Examination of Kap114p and yTBP levels in yeast strains under different experimental conditions. a, A representative spot assay result showing growth of WT, single or double gene knockout strains of *KAP114* (Δ*kap114*) and *TAF1-TAND* (Δ*taf1-tand*) serially diluted (1:5) on minimal medium

plates and incubated at 30 or 37 °C. The assays were performed in triplicate. **b,c,** BY4741 strains with or without expression of GFP-yTBP were grown in the absence (**b**) or presence (**c**) of 1.5 M NaCl and incubated at 30 °C. Cell density at OD_{600} nm was measured every day. Data are represented as mean ± SD (n=3 independent experiments). **d,e,** RT-qPCR analysis of genes transcribed by RNA Pol I (*RDN58*), RNA Pol III (*SNR6*), or RNA Pol II (TFIID-dependent *RPS5*, *RPS8A*, *RPS3*; SAGA-dependent *PYK1* and *PGK1*) in the BY4741 strains with or without expression of GFP-yTBP and grown at 30 °C in the absence (**d**) or presence (**e**) of 1.5 M NaCl. The bar graphs show the relative levels of indicated gene transcripts, normalized to *ACTIN* as the internal control. Data are represented as mean ± SD (n=3 independent experiments). **f,g,** The *KAP114* knockout strains expressing GFP-yTBP and wild-type *KAP114* or *KAP114*Δ(899-956) grown in minimal media without (**f**) or with (**g**) 1.5 M NaCl supplementation were analyzed by Western blot using GFP antibody. GAPDH was used as the control in Western blots. **h,** Expression of GFP-tagged yTBP in the *KAP114* knockout strains rescued by wild-type *KAP114* or *KAP114*Δ(899-956) and grown in minimal media with or without 1.5 M NaCl supplementation. The bar graphs show the band intensity ratio of GFP-yTBP to GAPDH. Data are represented as mean ± SD (n=6 independent experiments). Differences were assessed statistically by two-tailed Student's t test; n.s.; not significant. **i,j,** RT-qPCR analysis of gene expression by *KAP114* knockout strains expressing (**i**) *yTBP* and (**j**) wild-type *KAP114* or *KAP114*Δ(899-956). Yeast cells were grown in minimal media with or without 1.5 M NaCl supplementation. The bar graphs show the relative levels of *yTBP* and *KAP114* transcripts, normalized to *ACTIN* as the internal control. Data are represented as mean ± SD (n=3 independent experiments). N.A.: non-applicable.

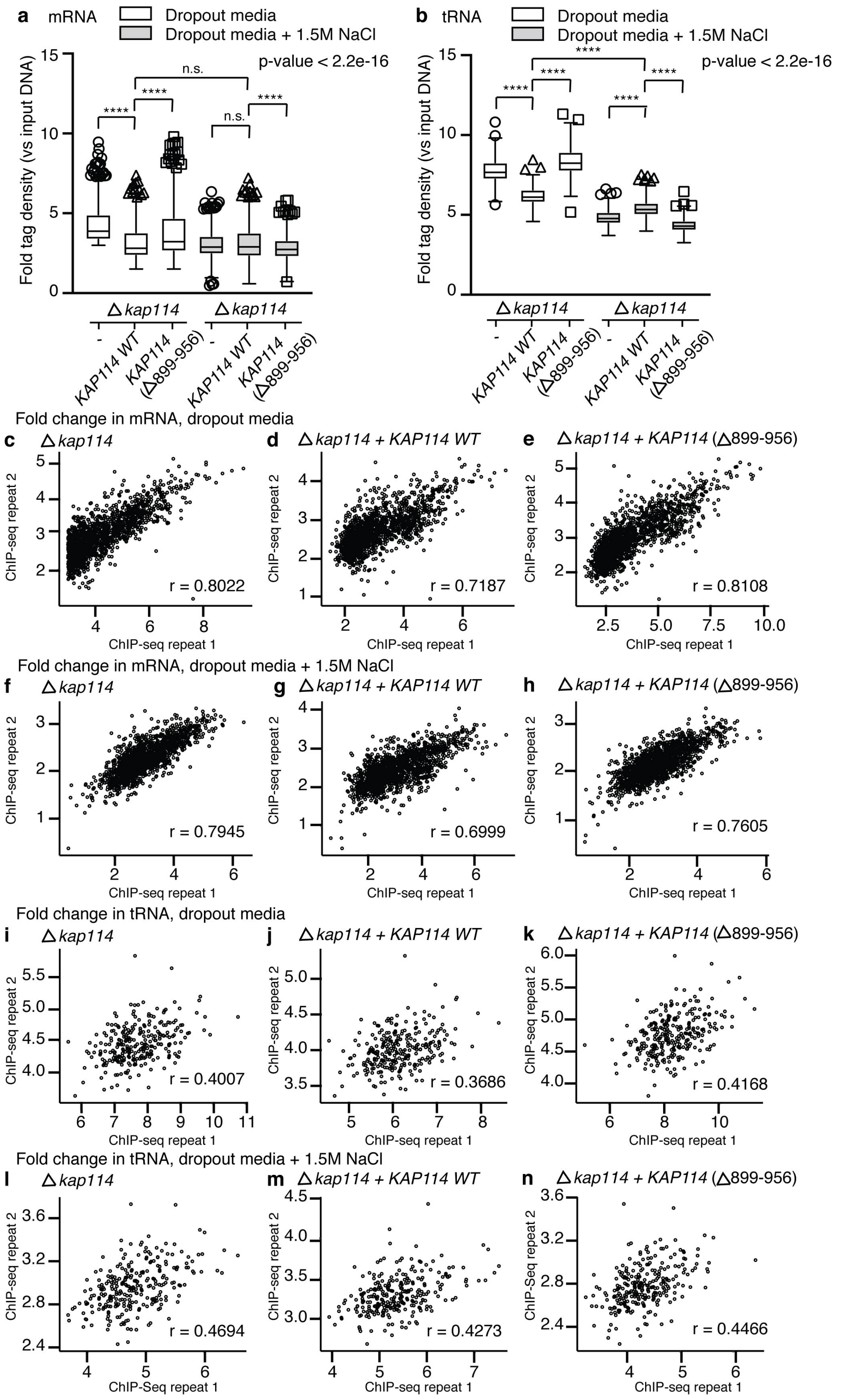

a mRNA
Dropout media
Dropout media + 1.5M NaCl
p-value < 2.2e-16
Fold tag density (vs input DNA)
n.s.
****
Δ kap114
-
KAP114 WT
KAP114 (Δ899-956)
b tRNA
Fold change in mRNA, dropout media
c Δ kap114
d Δ kap114 + KAP114 WT
e Δ kap114 + KAP114 (Δ899-956)
ChIP-seq repeat 1
ChIP-seq repeat 2
r = 0.8022
r = 0.7187
r = 0.8108
Fold change in mRNA, dropout media + 1.5M NaCl
f Δ kap114
g Δ kap114 + KAP114 WT
h Δ kap114 + KAP114 (Δ899-956)
r = 0.7945
r = 0.6999
r = 0.7605
Fold change in tRNA, dropout media
i Δ kap114
j Δ kap114 + KAP114 WT
k Δ kap114 + KAP114 (Δ899-956)
r = 0.4007
r = 0.3686
r = 0.4168
Fold change in tRNA, dropout media + 1.5M NaCl
l Δ kap114
m Δ kap114 + KAP114 WT
n Δ kap114 + KAP114 (Δ899-956)
r = 0.4694
r = 0.4273
r = 0.4466
ChIP-Seq repeat 1
ChIP-Seq repeat 2

Supplementary Figure 7|Validation of ChIP-based analysis by Kruskal-Wallis test. a,b, A box-whisker plot showing the fold-change of TBP ChIP-seq signals, relative to input DNA, at the promoters of mRNA (n=1,480) (**a**) or tRNA (n= 272) (**b**) genes for *KAP114* knockout strains rescued by vector control (-), wild-type *Kap114* or *Kap114*Δ(899-956) grown in the absence or presence of 1.5 M NaCl. The Kruskal-Wallis test with Dunn´s post-hoc test was used to determine the significance of each group. The *p* values from the Kruskal-Wallis test for each group are shown. Significant pairwise comparisons are annotated by an asterisk. n.s.: not significant, ****: p <0.0001. Box limits: 25–75% quantiles, middle: median, upper (lower) whisker to the largest (smallest) value. **c-n,** Scatterplots to illustrate the correlation in fold-change for pair-wise samples of ChIP-seq repeats 1 and 2 under regular and high-salt conditions. Pearson correlation coefficients (r) are indicated, supporting that the ChIP-seq results display high (mRNA) or moderate (tRNA) reproducibility.

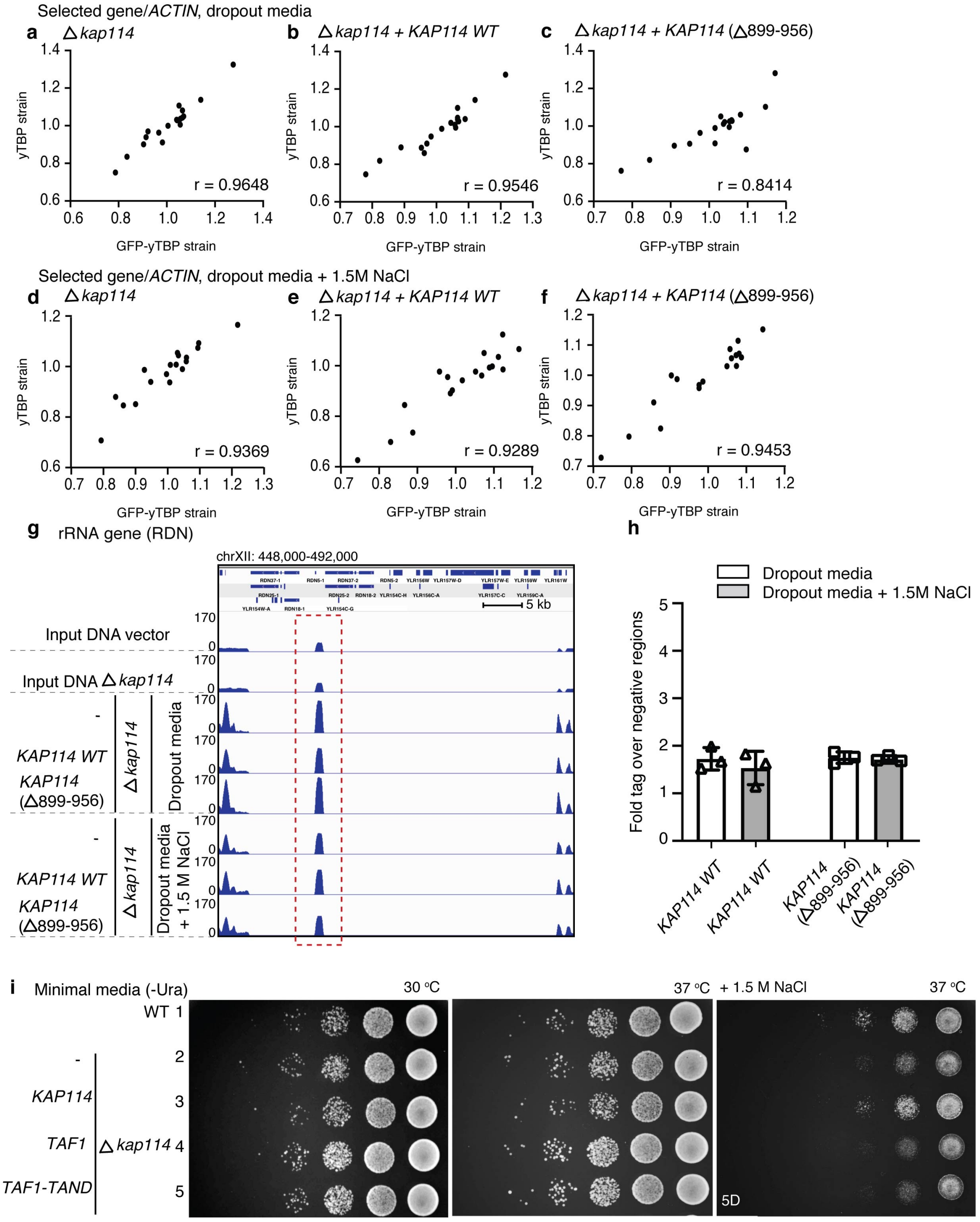

Supplementary Figure 8|ChIP-based analysis of the rRNA gene promoter affected by Kap114p. a-f, Scatterplots to illustrate the correlation in RT-qPCR results for selected genes (18 genes in total; Extended Data Table 2) normalized by *ACTIN* in yeast strains expressing yTBP with or without a GFP tag and under regular or high-salt conditions. Pearson correlation coefficients (r) are indicated. **g,** Gene tracks showing the TBP ChIP signals at the promoter of the *RDN5-1* gene in yeast chromosome XII:459,676-459,796. Wild type and *KAP114* knockout strains

rescued by vector control (-), wild type *KAP114* and *KAP114*Δ(899-956) were examined under regular and high salt conditions. The scale bar is indicated. **h**, Quantification of GFP-Kap114 ChIP-qPCR signal using anti-GFP antibody in the chromatin lysates of *KAP114* knockout strains expressing GFP-tagged wild type *KAP114* or *KAP114*Δ(899-956) under regular and high salt conditions. Data are presented as mean ± SD (n=3 biologically independent experiments). **i,** A representative result showing wild type and *KAP114* knockout strains rescued by vector control (-), wild-type *KAP114, TAF1* or *TAF1-TAND* and that were diluted serially (1:5), spotted onto minimal medium plates and incubated at 30 or 37 °C, or spotted onto minimal medium plates with 1.5 M NaCl and incubated at 37 °C. The assays were performed in triplicate.

Supplementary Table 1|Summary of Kap114p yTBP^{C} binding affinity by ITC

High concentration (50 μM Kap114p: 500 μM yTBP^{C})

**Kap114p•yTBP^{C} interaction**					
Kap114p	yTBP^{C}	Kd (nM)	Δ*H* (kCal/mol)	Δ*S* (Cal/mol.K)	Note
Wild type	Wild type	1.0	10.83	81.4	
**HEAT8B + loop**					
Y370A	Wild type	20.0	12.10	75.8	Y370 contacts N91 (yTPB)
Wild type	N91A	28.9	5.73	53.8	
Y370A	N91A	1500	16.19	81.0	
**HEAT13B**					
Wild type	R141A	255.0	10.99	67.2	R141 contacts N591 (Kap114p)
Wild type	K145A	237.8	16.19	87.1	K145 contacts D547 (Kap114p)
Q597A	Wild type	7.4	5.57	56.0	R137 contacts Q597 (Kap114p)
Q597A	R141A	316.0	7.53	55.1	
**HEAT19 loop**					
Wild type	ΔY933	7.7	12.06	77.7	Hydrophobic concave surface,
Wild type	ΔY939	37.1	15.71	87.0	including F99, L114 and F116
Wild type	Δ(a.a. 931-941)	158.0	13.16	75.3	(yTBP^{C})
**HEAT18-19 loop**					
Wild type	Δ(a.a.848-875)	1.7	7.73	66.1	

**Kap95p•yTBP^{C} interaction**				
Kap95p	yTBP^{C}	Kd (nM)		
Wild type	Wild type	8100	6.64	46.7
Wild type	R141A	9700	6.49	45.2

Low concentration (25 μM Kap114p: 250 μM yTBP^{C})

**Kap114p•yTBP^{C} interaction**				
Kap114p	yTBP^{C}	Kd (nM)	Δ*H* (kCal/mol)	Δ*S* (Cal/mol.K)
Wild type	Wild type	1.4	8.49	69.0
**HEAT8B + loop**				
Y370A	N91A	938	3.32	38.7
**HEAT13B**				
Q597A	R141A	165.3	9.10	62.7
**HEAT19 loop**				
Wild type	Δ(a.a. 931-941)	79.9	12.08	73.0

Supplementary Table 2| Primers

ChIP-qPCR

Gene Name	Forward	Reverse
YOR383C (FIT3)	TCTCAGCAAGAGCAGTAGCG	ACAAACAAGTACTTCCACTCGC
YHL047C (ARN2)	ACGCTTTGTTTGGCTAGAACG	TGTGTCCTTTCCACTTGAGC
YOR382W (FIT2)	GAATGTGTGCGTGCTTCAGG	TGCCGAGACGGCAGTCATAA
YMR319C (FET4)	ACCGAATTCTTCGTACTGTTTGC	TCTCGGGAATCCAGGTGCTA
YKL163W (PIR3)	TGTCTAACACTGAAACTGTAACCT	TAGCAGCTAAAGCGGAGACG

RT-qPCR

Gene Name	Forward	Reverse
YER148W (yTBP)	GTGGCAACTGTGACTTTGGGG	AATACGCATGATGACAGCAGC
YGL241W (Kap114)	TCTCTACTGAAGATGAGACC	GGCTTCCAAAATTCTTCCTA
YNCL0014C (RDN58)	AACGGATCTCTTGGTTCTCG	GTGCGTTCAAAGATTCGATG
YNCL0006W(SNR6)	CGAAGTAACCCTTCGTGGAC	TCATCCTTATGCAGGGGAAC
YNL178W (RPS3)	TACGGTGTCGTCAGATACG	GACCAGAGTGAATCAAGAAACC
YJR123W (RPS5)	GGATGCTTCTTTGGTTGACTAC	GGACATTGAGCCTTTCTGAATCTC
YBL072C (RPS8A)	AAAGATCCGCTACCGGTGCCAAG	TCTTGGAGATACCTTCAGAAGCCC
YCR012W (PGK1)	TGCTGCTTTGCCAACCATC	GTGACATCCTTACCCAACAATG
YAL038W (PYK1)	CCAACCTCCACCACCGAAAC	GGGCTTCAACATCATCAGTCCA
YGL030W (RPL30)	TCATTGCCGCTAACACTCCA	ACTTACCGACAGCAGTACCC
YDR037W (KRS1)	CCATTTGACCAAAGGGCACG	ATACCACAACCCCAACCACC
YLR060W (FRS1)	TACCGTCTCCGTGAACAAGC	GCCTCCTCTGTGGTGTCTTC
YJL190C (RPS22A)	CCGTGCTTTTGCATAACTTGC	GCTGAAAAGACCGGTAAGCG
YDR447C(RPS17B)	GAGCGGAAACGTTGATGACA	CCCAGAAGTCTCTGCTTTGGA
YKL006W(RPL14A)	CCGATTCTATTGTCAAGGCTTCT	CACCAGCTTTTGGACCGTCA
TEF4 (YKL081W)	GCCAAAGCACCCATTGGAAG	GCAAAGCAACTGGTCTGGTG
TIF1 (YKR059W)	GACACCTCTGTCAAGGCTCC	CCATGTGGAAAGCCAAAGCC
ASC1 (YMR116C)	GGTGCTTACGCTTTGTCTGC	ACATCGGACTTGTGACCGAC
FIT2 (YOR382W)	GTGTCGAGTGTAATGACCAC	GACAACGACAGTTCTAGTGG
FIT3 (YOR383C)	CTTGCTGAGAGTATCACCAC	TTTCGGCGGCAGAAGTTTC
TDH1 (YJL052W)	CCAAGAAGGTTGTCATCACT	TTGATAACCTTGGCCAATGG
FET3 (YMR058W)	CCGATACGACGTATCTTTTG	CCAGGACTGTATATCTCTGA
WSC4 (YHL028W)	CACAAGTACCTCATCGTCGA	CGACGTCGTGCTTGTAGAAA
HOR7 (YMR251W-A)	TCTCAAGTTGTTGTTTCCGC	AAAGCACCAGCTAGAGCAAC
ENO1 (YGR254W)	AGTCCAAGACCTCTCCATAC	CAATTCTCAAAGCTTCAGCG
SED1 (YDR077W)	CGGTACTTCTACTGAAGCTC	CAGTGACTACAGTGTAGTCAG
NCW1 (YMR122W-A)	TTTCCAACAATGTCGTTGCC	GGCCATGATGATACCGTACT

Supplementary Table 3|Yeast strains used in this study

Strain	Genotype	Source
YTK12029	*MATa his3Δ1 leu2Δ0 ura3Δ0 met15Δ0 Δtaf1::kanMX6 pM4770/TAF1*	A gift from Dr. Tetsuro Kokubo[6,7]
YTK12803	*MATa his3Δ1 leu2Δ0 ura3Δ0 met15Δ0 Δtaf1::kanMX6 pM7121/taf1ΔTAND*	A gift from Dr. Tetsuro Kokubo[6,7]
BY4741	*MATa his3Δ1 leu2Δ0 met15Δ0 ura3Δ0*	Taiwan Yeast Bioresource Center at the First Core Labs, National Taiwan University
YER148W-GFP	*MATa his3Δ1 leu2Δ0 met15Δ0 ura3Δ0 spt15- GFP(S65T)-His3MX*	Purchased from Invitrogen[8]
SC1005	*MATa his3Δ1 leu2Δ0 ura3Δ0 met15Δ0 Δtaf1::kanMX6 kap114::LEU2 pM4770/TAF1*	
SC1007	*MATa his3Δ1 leu2Δ0 met15Δ0 ura3Δ0 spt15- GFP(S65T)-His3MX kap114::LEU2*	
SC1008	*MATa his3Δ1 leu2Δ0 ura3Δ0 met15Δ0 Δtaf1::kanMX6 kap114::LEU2 pM4770/TAF1* pRS426/*KAP114*	
SC1010	*MATa his3Δ1 leu2Δ0 ura3Δ0 met15Δ0 Δtaf1::kanMX6 kap114::LEU2 pM4770/TAF1* pRS426/*KAP114(Δ899-956)*	
SC1011	*MATa his3Δ1 leu2Δ0 ura3Δ0 met15Δ0 Δtaf1::kanMX6 kap114::LEU2 pM4770/TAF1* pRS426/*MOT1*	This study
SC1012	*MATa his3Δ1 leu2Δ0 ura3Δ0 met15Δ0 Δtaf1::kanMX6 kap114::LEU2 pM4770/TAF1* pRS426/*TAF1*	This study

SC1013	*MATa his3Δ1 leu2Δ0 ura3Δ0 met15Δ0 Δtaf1::kanMX6 Kap114::LEU2 pM4770/TAF1* pRS426/*TAF1(TAND)*	This study
SC1014	*MATa his3Δ1 leu2Δ0 ura3Δ0 met15Δ0 Δtaf1::kanMX6 kap114::LEU2 pM4770/TAF1* pRS426/*TAF1(ΔTAND)*	This study
SC1115	*MATa his3Δ1 leu2Δ0 ura3Δ0 met15Δ0 Δtaf1::kanMX6 pM7121/taf1ΔTAND kap114::LEU2*	This study
SC1116	*MATa his3Δ1 leu2Δ0 met15Δ0 ura3Δ0 spt15- GFP(S65T)-His3MX kap114::LEU2* pRS426/*KAP114*	This study
SC1117	*MATa his3Δ1 leu2Δ0 met15Δ0 ura3Δ0 spt15- GFP(S65T)-His3MX kap114::LEU2* pRS426/ *KAP114(Δ899-956)*	This study

Supplementary Table 4|Plasmids used in this study

Plasmid	Encoded protein
**Bacterial expression construct**	
pGEX-6P-1-*Kap114*	GST-Kap114p
pGEX-6P-1-*Kap95*	GST-Kap95p
pGEX-6P-1-*Kap121*	GST-Kap121p
pET28a-Ran (Q69L)	His-Ran (Q69L)
pGEX-6P-1-*Spt15 (61-240)*	GST-yTBP (61-240)
pGEX-6P-1-*Kap114 (Δ347-371)*	GST-Kap114p (Δ347-371)
pGEX-6P-1-*Kap114(Δ899-956)*	GST-Kap114p (Δ899-956)
pGEX-6P-1-*Kap114(899-956)*	GST-Kap114p (899-956) /HEAT19 loop
pGEX-6P-1-*Kap114 (Y370A)*	GST-Kap114p (Y370A)
pGEX-6P-1-*Kap114 (Q597A)*	GST-Kap114p (Q597)
pGEX-6P-1-*Kap114 (Δ933)*	GST-Kap114p (Δ933)
pGEX-6P-1-*Kap114 (Δ939)*	GST-Kap114p (Δ939)
pGEX-6P-1-*Kap114 (Δ933, Δ939)*	GST-Kap114p (Δ933, Δ939)
pGEX-6P-1-*Kap114 (Δ932-941)*	GST-Kap114p (Δ932-941)
pGEX-6P-1-*Kap114 (ΔHEAT18-19Loop)*	GST-Kap114p (Δ848-875)
pET28a-*Spt15 (61-240)*	His-yTBP (61-240)
pET28a-*Spt15 (61-240) (N91A)*	His-yTBP (61-240) (N91A)
pET28a-*Spt15 (61-240) (R141A)*	His-yTBP (61-240) (R141A)
pET28a-*Spt15 (61-240) (K145A)*	His-yTBP (61-240) (R145A)
**Yeast expression construct**	
pRS426-*KAP114*	Kap114p
pRS426-*KAP114 (Δ347-371)*	Kap114p (Δ347-371)
pRS426-*KAP114 (Δ899-956)*	Kap114p (Δ899-956)
pRS426-*MOT1*	MOT1
pRS426-*TAF1*	TAF1
pRS426-*TAF1 (TAND)*	TAF1 (1-71)
pRS426-*TAF1 (ΔTAND)*	TAF1 (72-End)

Supplementary Table 5| Summary of number of reads detected in ChIP-Seq assays.

Sample	Total reads	Uniquely aligned reads	Mapping efficiency (%)
**Data presented in Extended Data Fig. 6h,i**			
SC1005_Input DNA	13,710,185	11,303,884	82.45
SC1005_reKap114WT_Input DNA	14,011,499	10,737,868	76.64
SC1005_GFPyTBP_TBPChIP	14,884,603	10,826,238	72.73
SC1005_GFPyTBP_TBPChIP_Salt	13,526,361	9,702,049	71.73
SC1005_reKap114WT_GFPyTBP_TBPChIP	13,004,304	9,693,167	74.54
SC1005_reKap114WT_GFPyTBP_TBPChIP_Salt	12,842,503	8,147,074	63.44
SC1005_reKap114delta_GFPyTBP_TBPChIP	9,460,109	6,983,034	73.82
SC1005_reKap114delta_GFPyTBP_TBPChIP_Salt	12,324,552	9,100,612	73.84
**Data presented in Fig. 5e,f**			
SC1005_Input DNA	38,422,922	30,550,876	79.51
SC1005_GFPyTBP_TBPChIP	36,034,182	25,453,071	70.64
SC1005_GFPyTBP_TBPChIP_Salt	35,493,983	25,396,505	71.55
SC1005_reKap114WT_GFPyTBP_TBPChIP	40,179,369	28,747,841	71.55
SC1005_reKap114WT_GFPyTBP_TBPChIP_Salt	36,168,778	25,992,657	71.86
SC1005_reKap114delta_GFPyTBP_TBPChIP	36,655,961	25,507,372	69.59
SC1005_reKap114delta_GFPyTBP_TBPChIP_Salt	40,231,432	29,197,498	72.57

Supplementary References

1 Zivanov, J. *et al.* New tools for automated high-resolution cryo-EM structure determination in RELION-3. *Elife* **7**, doi:10.7554/eLife.42166 (2018).

2 Zheng, S. Q. *et al.* MotionCor2: anisotropic correction of beam-induced motion for improved cryo-electron microscopy. *Nat Methods* **14**, 331-332, doi:10.1038/nmeth.4193 (2017).

3 Punjani, A., Rubinstein, J. L., Fleet, D. J. & Brubaker, M. A. cryoSPARC: algorithms for rapid unsupervised cryo-EM structure determination. *Nat Methods* **14**, 290-296, doi:10.1038/nmeth.4169 (2017).

4 Sievers, F. *et al.* Fast, scalable generation of high-quality protein multiple sequence alignments using Clustal Omega. *Mol Syst Biol* **7**, 539, doi:10.1038/msb.2011.75 (2011).

5 McGuffin, L. J., Bryson, K. & Jones, D. T. The PSIPRED protein structure prediction server. *Bioinformatics* **16**, 404-405, doi:10.1093/bioinformatics/16.4.404 (2000).

6 Anandapadamanaban, M. *et al.* High-resolution structure of TBP with TAF1 reveals anchoring patterns in transcriptional regulation. *Nat Struct Mol Biol* **20**, 1008-1014, doi:10.1038/nsmb.2611 (2013).

7 Ohyama, Y., Kasahara, K. & Kokubo, T. Saccharomyces cerevisiae Ssd1p promotes CLN2 expression by binding to the 5'-untranslated region of CLN2 mRNA. *Genes Cells* **15**, 1169-1188, doi:10.1111/j.1365-2443.2010.01452.x (2010).

8 Huh, W. K. *et al.* Global analysis of protein localization in budding yeast. *Nature* **425**, 686-691, doi:10.1038/nature02026 (2003).
